# Reconfigurable memlogic long wave infrared sensing with superconductors

**DOI:** 10.1038/s41377-024-01424-2

**Published:** 2024-04-26

**Authors:** Bingxin Chen, Huanyi Xue, Hong Pan, Liping Zhu, Xiaomi Yan, Jingzhe Wang, Yanru Song, Zhenghua An

**Affiliations:** 1 State Key Laboratory of Surface Physics and Institute for Nanoelectronic Devices and Quantum Computing, Department of Physics, Fudan University, Shanghai, 200438 China; 2 ShanghaiTech Quantum Device Lab, ShanghaiTech University, Shanghai, 201210 China; 3 Shanghai Qi Zhi Institute, 41th Floor, AI Tower, No. 701 Yunjin Road, Xuhui District, Shanghai, 200232 China; 4 Yiwu Research Institute of Fudan University, Chengbei Road, Yiwu City, 322000 Zhejiang China; 5 Zhangjiang Fudan International Innovation Center, Fudan University, Shanghai, 201210 China

**Keywords:** Imaging and sensing, Photonic devices, Nanophotonics and plasmonics, Optical sensors, Optoelectronic devices and components

## Abstract

Optical sensors with in-cell logic and memory capabilities offer new horizons in realizing machine vision beyond von Neumann architectures and have been attempted with two-dimensional materials, memristive oxides, phase-changing materials etc. Noting the unparalleled performance of superconductors with both quantum-limited optical sensitivities and ultra-wide spectrum coverage, here we report a superconducting memlogic long-wave infrared sensor based on the bistability in hysteretic superconductor-normal phase transition. Driven cooperatively by electrical and optical pulses, the device offers deterministic in-sensor switching between resistive and superconducting (hence dissipationless) states with persistence > 10^5^ s. This results in a resilient reconfigurable memlogic system applicable for, e.g., encrypted communications. Besides, a high infrared sensitivity at 12.2 μm is achieved through its in-situ metamaterial perfect absorber design. Our work opens the avenue to realize all-in-one superconducting memlogic sensors, surpassing biological retina capabilities in both sensitivity and wavelength, and presents a groundbreaking opportunity to integrate visional perception capabilities into superconductor-based intelligent quantum machines.

## Abstract (web summary)

We demonstrate a novel superconducting LWIR sensor integrating the function of infrared sensitivity, memory and reconfigurable logic computing.

## Graphical abstract

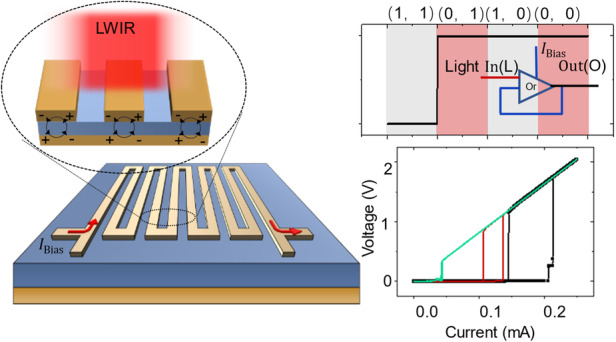

## Introduction

The rapid advancement of artificial intelligence (AI) and the Internet of Things (IoT) has sparked a significant increase in optical sensory nodes generating copious unstructured raw data^1,2^. This surge occurs not only in the visible light spectrum, where machine vision is pivotal for intelligent systems, but also in the invisible spectrum, notably the infrared region^3,4^. In this invisible realm, machine perception plays a crucial role across a wide array of applications such as thermal imaging, surveillance, industrial monitoring, gas detection, medical thermography, defense and space exploration etc.^5–7^. In the visible range, inspired by the intelligent biological approach of the retina’s neurons, which not only detect light stimuli but also engage in initial image processing, memlogic optical sensing or in-sensor computing emerges and provides a promising route to address the sensing and processing bottleneck^8^. These innovative works employ novel materials such as two-dimensional material^9–11^, metal-oxide memristors^12^, phase-change materials (PCMs) ^13^ etc and have shown significantly reduced power consumption, reduced time delays, and minimized hardware redundancy. More remarkably, unprecedentedly new functionalities have also been demonstrated such as supervised and unsupervised learning and training, highlighting the compelling advantages of memlogic sensors over traditional ones^14^. Conversely, in the infrared spectrum, this groundbreaking innovation has unfortunately received little attention, despite the fact that this spectral region is invisible only to humans but should not be to machines in the forthcoming intelligent era^15^.

In the pursuit of developing memlogic sensors for the infrared spectrum, it is crucial to explore alternative materials with memory capabilities and meanwhile sensitivity to infrared radiation. Superconductors stand out as promising candidates not only due to their extraordinary photo-sensitivities^16^ but also because of their remarkable ability to undergo sharp phase transitions, akin to the characteristics of phase-change random access memory (PCRAM), which is celebrated for its rapid data access, durability, and non-volatile attributes^17^. Recent advancements in non-von Neumann superconducting neuromorphic circuits have made significant strides, employing various principles, including quantum-phase slip junctions^18^, Josephson junctions^19^, magnetic Josephson junctions^20^, and superconducting nanowires^21^. These innovations have showcased superior memory effects and enhanced processing speed while minimizing energy consumption. Concerning optical sensing capabilities, superconductors also excel, as evidenced by technologies such as Microwave Kinetic Inductance Detectors (MKIDs), Superconducting Nanowire Single Photon Detectors (SNSPDs), and Transition-Edge Sensors (TESs). These superconducting detectors offer an exceptionally wide spectral coverage including but not limited to the infrared, spanning from microwaves to X-rays, and deliver unparalleled sensitivity, outperforming other photodetection technologies. However, despite these promising attributes, there have been limited coordinated efforts to fully harness these typically independent advantages of superconductors, particularly in the context of creating memlogic sensors, especially for the infrared spectrum.

Here we report for the first time a superconducting memlogic sensor that integrates the capabilities of infrared sensitivity, memory retention, and reconfigurable logic computing, all-in-one infrared memlogic sensory. We demonstrate the simultaneous four controls of this superconducting device including optical/electrical biases, and optical/electrical pulses allow different encoding logic with increasing functional complexity. The exemplified wavelength $\lambda \sim 12.2\,{\rm{\mu }}{\rm{m}}$ lies in the important atmospheric infrared window ($8 \sim 14\,{\rm{\mu }}{\rm{m}}$) as favored for many applications. Distinctive from existing superconducting sensors (like SNSPDs and TESs), our device works in the bistable region of the hysteretic superconductor-normal phase transition. When subjected to an appropriate optical or electrical pulse, the device transitions from a superconducting state to a normal one. Notably, this normal state remains memorized even after the optical or electrical pulse is turned off, due to the self-heating effect within the resistive state which is reminiscent of PCRAM. Driven cooperatively by electrical and optical pulses, the device exhibits deterministic in-sensor switching between resistive and superconducting states with persistence >10^5^ s. As a result, our sensor can be programmed with both optical and electrical spikes, enabling electronic in-memory computing, in-sensor computing, optical remote programming and hence all-in-one single devices. The versatility of reconfigurable logic operations, including “AND”, “OR”, “signal follower” and more intricate logic operations are demonstrated in our single memlogic sensor. This is distinctive from previous approach^22^ that used different superconducting subcells for optical sensing and computing functions, respectively. With these reconfigurable operations, we show the infrared remote encrypted communication at a single device level. To overcome the well-known low system detection efficiency of superconducting detection in this long-wave infrared region^23–25^, a metasurface^26^ perfect absorber concept is employed which consists of a resonant plasmonic cavity, with tri-layer structure of Nb-Si-Nb. Given the prevalence of bistable effects, extensive wavelength coverage, and high photo-sensitivity in superconductors, our memlogic sensing concept, utilizing superconducting phase transitions, shows potential for versatile applications across a wide electromagnetic spectrum and down to quantum-levels in various fields such as machine perception, remote sensing, secure communication, and space detection. In addition, our work may also find potential application with the development of low temperature quantum computing, where efficient conversion of optical to electric signal is a standing item on the agenda.

## Results

### Electrical controlling bistability

**Fig. 1:**
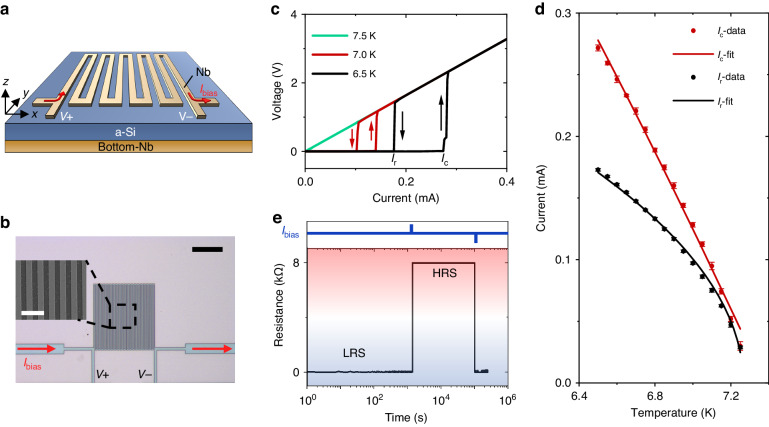
Characterization of electrical controlling bistability. **a** Schematic depiction of a devices **b** Optical micrograph of pseudo-color memlogic sensor. Four-probe method is used with constant current source meter during the measurement. Scale bar $50\,{\rm{\mu}}{\rm{m}}$. Inset: SEM image of part of photosensitive area. Scale bar $5\,{\rm{\mu}}{\rm{m}}$. **c** Different I-V hysteretic curves under various temperature. **d** Critical current and retrapping current change with temperature. **e**, Retention and electrical switching of HRS and LRS at $6.5\,{\rm{K}}$. A high pulse current ($400\,{\rm{\mu }}{\rm{A}}$, duration $0.3\,{\rm{s}}$) is used for the set process, and the reset process is initiated by low pulse current ($1\,{\rm{\mu }}{\rm{A}}$, duration $0.3\,{\rm{s}}$), reading with current of $200\,{\rm{\mu }}{\rm{A}}$

**Fig. 2:**
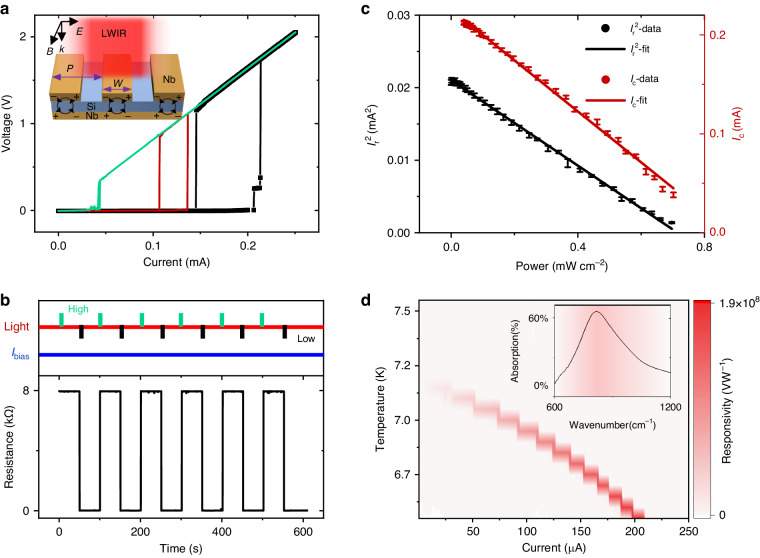
Optical controlling bistability. **a** Different I-V hysteretic curves under the illumination of infrared light ($820\,{{\rm{cm}}}^{-1}$) with different intensity at $6.5\,{\rm{K}}$. The green, red and black curves represent the light intensity of $0.66\,{\rm{mW}}\,{{\rm{cm}}}^{-2}$ (light 2), $0.34\,{\rm{mW}}\,{{\rm{cm}}}^{-2}$ (light 1) and 0 (light 0), respectively. Inset: Schematic depiction of a plasmonic absorber. **b** Resistance switching through light pulse (duration of $0.3\,{\rm{s}}$), read at current of $125\,{\rm{\mu }}{\rm{A}}$. **c** The experimental data and fitting model for retrapping current and critical current, which decrease as the light power increases. **d** The temperature and current dependence of responsivity. The lower temperature, the higher responsivity. Inset: Absorption spectrum of the sensor shows the metamaterials resonance frequency at $820\,{{\rm{cm}}}^{-1}$ (wavelength $12.2\,{\rm{\mu }}{\rm{m}}$)

Figure 1a, b displays the superconductor Nb sensor used in this study, which consisted of a grating wire with a width $W=1.3\,{\rm{\mu }}{\rm{m}}$, thickness of $h=60\,{\rm{nm}}$ and period of $P=2.5\,{\rm{\mu }}{\rm{m}}$. The device features a meander-shaped design, allowing for electrical measurements via four electrical contacts. As shown in Fig. 1c, we study the hysteretic current-voltage characteristic (IV) without intentional light illumination at different temperatures between 6.5 and 7.5 K. The critical temperature of our device is about 7.3 K (see Supplementary Note 1, Supplementary Fig. S1a). In a hysteretic IV curve, when the current is ramped up from zero, the device typically switches to a non-zero voltage state (i.e., resistive state) at the critical current ${I}_{c}$. The subsequent current ramp down gives a switching to zero-voltage state at a smaller current, called retrapping current ${I}_{r}$. In this cycle, the hysteric behavior is predominantly ascribed to thermal origin^27^, similar to PCRAM in which Joule heating is one of key mechanisms for operation. This is in contrast to tunnel-barrier type Josephson junctions in which hysteresis arises from large junction capacitance^28^. To investigate the temperature dependence of our device’s critical current ${I}_{c}$ and retrapping current ${I}_{r}$, hysteretic loops at different temperature are recorded as shown in Fig. 1c. It can be seen that the hysteretic loop become smaller with increasing the temperature until reaching the critical temperature (*T*_*c*_). Figure 1d displays the measured ${I}_{c}$, ${I}_{r}$ as functions of temperature. We find that the critical current follows the expression ${I}_{c}\approx I(0)\left[1-{(T/{T}_{C})}^{2}\right]$ from Silsbee’s rule^29^ instead of Bardeen’s phenomenological expression or Ginsbury-Landau (GL) theory as depicted by the red line in Fig. 1d. This implies that Nb wire in our device is more like three-dimensional as both the width and the thickness of our Nb wire are both larger than coherent length and penetration depth (~40 nm). As a reference, superconducting wires in SNSPDs are often quasi-one-dimensional. From above, we obtain $I\left(0\right)=1.24\,{\rm{mA}},{T}_{C}=7.38\,{\rm{K}}$, which represent the critical current at zero temperature and critical temperature, respectively. The ${T}_{C}$ value is consistent with the direct experimental observation of superconducting transition with low driving current. Despite the microscopic origin being very sophisticated, the agreement with Silsbee’s rule suggests that the destroy of superconductivity above ${I}_{c}$ may be related to the self-field effect of the supercurrent which thereby induces vortex in the Nb wire and changes it into the dissipative intermediate state. Besides, signatures of phase slip fluctuations have also been observed, as indicated by the voltage step in Fig. 1c black line and Fig. 2a black line. This suggests that phase-slip centers (PSCs) may appear with increasing current and play an important role in the competing processes of the superconducting-to-normal phase transition^30,31^: superconducting (dissipationless) current flow and energy dissipation occurring simultaneously due to interactions with Andreev quasiparticles^30^. These PSCs could originate from sample defects or inhomogeneities due to, e.g., nanolithography processes. We note that, however, PSCs are not dominant and will not affect our memlogic operation as the phase-slip voltage steps are found to be very rare and exit only at lowest temperature (Fig. 1c).

In backward scan of the driving current, the device cannot return to the superconducting state until the heat generated by the current self-heating effect is smaller than the heat transferred to the substrate, corresponding to a threshold retrapping current ${{\rm{I}}}_{r}$. We find that this retrapping current agrees well with previous hotspot model^27^ and can be expressed by ${I}_{r}={(\alpha {W}^{2}{T}_{c}d/\rho )}^{1/2}{(1-T/{T}_{C})}^{1/2}$, where $\alpha ,{W},{d},\rho$, are heat-transfer coefficient per unit area, width, thickness and resistivity of the device, respectively (Supplementary Note 5). From this formula and Fig. 1d, we can get $\alpha =5.86\,{\rm{W}}$ ${{\rm{cm}}}^{-2}\,{\rm{K}}^{-1},\,{T}_{C}=7.27\,{\rm{K}}$. The value of $\alpha$ agrees well with previous literature^32^, which ranges from 2 to 10 ${\rm{W}}\,{{\rm{cm}}}^{-2}\,{\rm{K}}^{-1}$ and ${T}_{C}=7.27\,{\rm{K}}$ is also reasonably consistent with our critical temperature measure data (Supplementary Fig. S1a) and the derived value from Silsbee expression of ${I}_{c}$. The excellent agreement of ${I}_{r}$ with the hotspot model suggests that the normal-to-superconducting transition in the backward current scan is predominantly of thermal origin. This is also confirmed by our independent numerical study (Supplementary Note 6). On the other hand, we mention that we take only the stead-state terms in the above hotspot model and also in numerical analysis. Therefore, the heat is rather globally distributed in our sample, similar to TESs, but different from the spatially localized hotspot in SNSPDs.

In the above bistable region, when a bias current ${I}_{{bias}}$ is applied between the ${I}_{r}$ and ${I}_{c}$ (${I}_{r} < {I}_{{bias}} < {I}_{c}$), the device can be operated either in the superconducting zero voltage state (also known as low resistance state, LRS) or normal non-zero voltage state (high resistance state, HRS), depending on both the previous and present states. As shown in Fig. 1e, initially, the device operates at LRS with a current bias of $200\,{\rm{\mu }}{\rm{A}}$ (${I}_{r} < {I}_{{bias}} < {I}_{c}$) at 6.5 K, then a high electrical pulse ($400\,{\rm{\mu }}{\rm{A}}$ > ${I}_{c}$, duration 0.3 s) switches the LRS to HRS. This HRS state can be preserved for more than ${10}^{5}$ seconds with no drift even when the current drops back to $200\,{\rm{\mu }}{\rm{A}}$ but is still larger than ${I}_{r}$. Similarly, a low electrical pulse ($30\,{\rm{\mu }}{\rm{A}} < {I}_{r}$, duration 0.3 s) switches the device from the HRS to LRS and maintains LRS with the same current bias of $200\,{\rm{\mu }}{\rm{A}}$. We experimentally find that these operations have excellent repeatability and reliability, demonstrating no error bits during 10^6^ cycles of switching between HRS and LRS, as shown in Supplementary Fig. S1b of Supplementary Note 1. The response time of writing and erasing is 2.2 ${\rm{\mu }}{\rm{s}}$ and 1.9 ${\rm{\mu }}{\rm{s}}$, respectively (Supplementary Fig. S1c in Supplementary Note 1). The above excellent state persistence and cycling endurance make our device a good memristor, though working at low temperatures. As a comparison, memristors based on other (room temperature) materials typically suffer from instability^33^, poor cycling endurance and filament rupture^34^ etc. Besides, the precisely controllable transitions between the subcritical and supercritical current states make the device very promising for memlogic optical sensing like biological neurons, as shown later.

### Optical controlling bistability

So far, for most of the reported superconducting devices, such as superconducting quantum interference devices (SQUID)^32^ and superconducting photodetectors^35^, this hysteretic IV is often considered as a hindrance to devices’ performance although they can be indeed utilized as switches or memories^36^. By contrast, here we take the advantages of the hysteresis behavior and show its favorable applications in memlogic optical sensing.

In order to achieve a high infrared absorption, the resonant plasmonic metamaterials absorber consists of two metallic elements: a 60 nm thick Nb grating resonator and 100 nm thick Nb ground plane, with a 280 nm thick Si dielectric layer spaced in between, as shown in the inset of Fig. 2a. The area of Nb grating resonator is $100\times 100\,{\rm{\mu }}{{\rm{m}}}^{2}$ with a typical length $L=100\,{\rm{\mu }}{\rm{m}}$, width $W=1.3\,{\rm{\mu }}{\rm{m}}$ and period $P=2.5\,{\rm{\mu }}{\rm{m}}$ and is connected in a meander-shape to allow electrical measurement. The Nb ground plane is thick enough to block the transmission of incident light. This three-layer metamaterial couples to both the magnetic and electric components of incident electromagnetic waves and allows for minimization of the reflectance^37^. As schematically shown in inset Fig. 2a, localized surface plasmons are excited along the short Nb wire axis when perpendicularly polarized light excites Nb wire. The plasmonic oscillation in upper Nb stripes causes an antiphase oscillating mirror counterpart in the Nb ground plane, resulting in a circular current and an effectively magnetic response. The plasmonic resonant frequency of our sensor is measured to be $820\,{\rm{c}}{{\rm{m}}}^{-1}(12.2\,{\rm{\mu }}{\rm{m}})$, as shown in inset Fig. 2d. This resonance can be flexibly designed with different cavity parameters (see Supplementary Note 3, Supplementary Fig. S3). To simplify, it can be considered as a horizontal Fabry-Perot-like resonant mode, whose first-order resonance wavelength is roughly given^38^ by ${\lambda }_{0}=2{n}_{{eff}}W$, where ${n}_{{eff}}$ is the effective refraction index of the mode in the absorber cavity of width W. The incident light is mainly absorbed by Nb wire due to the local plasmons (see Supplementary Note 2, Supplementary Fig. S2). The local electric field is enhanced resonantly in the close vicinity of Nb wire with oscillating current inside. Thus, the Copper-pair will be destroyed efficiently when the light hits the Nb wire. The absorption peak at resonant frequency reaches nearly 70% (Inset of Fig. 2d) and we can further enhance this peak absorption (see Supplementary Note 3, Supplementary Fig. S3c) by optimizing the geometry of metamaterials to the perfect absorption condition^39^. Consequently, this plasmonic structure enhances its efficiency in harvesting the target infrared light, which thereby triggers the superconducting phase transition with high sensitivities.

As shown in Fig. 2a, the IV cures are significantly affected by infrared light at its resonant frequency with different intensities. Therefore, using optical pulses with appropriate intensity, we can manipulate the bistability of the device (Fig. 2b). For this purpose, attenuated quantum cascade laser is employed for optical excitation due to its excellent wavelength tunability and directionality as well as controllability as optical stimulating pulses. At a constant electrical bias current of 125 ${\rm{\mu }}{\rm{A}}$, the device’s state is changed to HRS under the illumination of light 2 ($0.66\,{\rm{mW}}\,{{\rm{cm}}}^{-2}$, green line), reset to LRS upon illumination of light 0 (black line, indicating no illumination), and maintain its previous state upon illumination of light 1 ($0.34\,{\rm{mW}}\,{{\rm{cm}}}^{-2}$, red line). The response time of writing and erasing by light is 5.4 ms and 4.5 ms, respectively, as shown in Supplementary Fig. S4. This illustrates the optical memlogic function of the device. Under illumination of light 1, the device’s state can be either HRS or LRS, depending on the pre-existing memory state. It is noteworthy to mention that the device is designed to efficiently harvest the incident optical illumination and therefore the laser illumination introduces substantial heating effect which interplays with the electrical Joule heating effect, eventually enabling the optical and electrical encoding and memlogic functionalities. On the other hand, the laser heating effect plays different roles in affecting the two boundaries (${I}_{r}$ and ${I}_{c}$) of the bistable hysteresis as discussed in the following.

To reveal the physics underlying the optical controlling of bistability, we examine the light intensity dependence of ${I}_{r}$ and ${I}_{c}$. The effect of incident photons is to reduce the free-energy barrier of phase slips in the superconductor, thereby causing a proliferation of phase-slip events and leading to a superconducting-to-normal transition^40^. The reduction of free-energy barrier of phase slips caused by the incident light has the similar effect with increasing the temperature^40^. This reduces ${I}_{c}$, which is given by ${I}_{c}(P)={I}_{0}-{AP}$, where *P* is the light power density, ${I}_{0}$ is the critical current without light radiation, and A is the coefficient representing how significant light illumination affects the critical current^41^. Experimental data gives that ${I}_{0}=0.215\,{\rm{mA}},{\rm{and}}\,{A}=0.245\,{\rm{A}}\cdot {{\rm{W}}}^{-1}$. Our experimental data of ${I}_{c}(P)$ show the linear dependence on light power, which is agrees well with previous literature^41^. On the other hand, ${I}_{r}$ is determined by classical thermal balance. In addition to Joule heat, the incident light will induce an additional heating source. Hence, we assumed that ${{I}_{r}(P)}^{2}=\alpha {W}^{2}\left({T}_{c}-{T}_{b}\right)/{R}_{n}-\beta S/{R}_{n}\cdot P$, where $\beta$ is absorption coefficient of light, ${R}_{{\rm{n}}}$ is the sheet resistance in the normal state, *S* is the area of sensor, ${T}_{b}$ is the temperature of substrate. (see Supplementary Note 5). We take the value $\alpha =5.86\,{\rm{W}}\,{{\rm{cm}}}^{-2}\,{\rm{K}}^{-1}$ obtained previously from the Fig. 1d and then obtain $\beta =0.75,{T}_{c}=7.05\,{\rm{K}}$. The value of $\beta$ agrees reasonably with the measured peak absorption coefficient ($\sim \! 0.7$) as shown in Fig. 2d. The slightly larger value of the derived $\beta$ than measured peak value may arise from the unmatched measurement configurations in reflection and photoresponse experiments (e.g., slightly different light spot size and/or light angle dispersion). The obtained critical temperature ${T}_{c}=7.05\,{\rm{K}}$ is slightly lower than the measured critical temperature without light irradiation ($\sim 7.3\,{\rm{K}})$. This implies that the infrared illumination may possibly introduce additional microscopic features like phase-slips which influence the superconducting transition.

The excellent optical controllability o*f* ${I}_{c}(P)$ and ${I}_{r}(P)$ which define the boundaries of the bistable hysteric window offers large degree of freedom to modulate the inequality relation between the operating biasing current ${I}_{{bias}}$ and ${I}_{c}(P)$, ${I}_{r}(P)$. As an example, at a constant bias current of 125 μA (temperature is fixed at 6.5 K), different light intensities justify different set of ${I}_{{\rm{c}}}(P)$ and ${I}_{r}(P)$: For light 2, ${I}_{{bias}}$ > ${I}_{c}(P)$, so the free-energy barrier of phase slips is suppressed by incident photons, causing proliferation of phase-slip events and hence trigger the phase transition. Eventually, the device switches to HRS; Oppositely for light 0, ${I}_{{bias}}$ < ${I}_{r}(P)$, so the device switches to superconducting state (LRS) due to less Joule heating and optical pumping than dissipated energy to the substrate; For the moderate intensity Light 1, ${I}_{r}(P) <$ ${I}_{{bias}} <$ ${I}_{c}(P)$, the device lies in the bistable region with memory capability.

Because our sensor was operated at the constant current mode, following traditional infrared photodetectors, the responsivity of the device can be evaluated using the expression ${R}_{{Vc}}=\triangle V/P={(V}_{P}-{V}_{0})/P$, where the ${V}_{P}$ and ${V}_{0}$ represent the voltage of device under irradiation of infrared light with power $P$ and without intentional light illumination, respectively. When the device is operated in the superconducting state with ${I}_{{bias}} <$ ${I}_{c}(P)$, the voltage can be regarded as nearly zero. Therefore, ${R}_{{Vc}}$ = $({I}_{0}-{AP})R/P$, where R is the resistance of normal state. The responsivity is related to the temperature and illumination power, with lower temperatures leading to larger responsivities. As shown in Fig. 2d (see also Methods), our device demonstrates a high responsivity of $1.9\times {10}^{8}\,{\rm{V}}{{\rm{W}}}^{-1}$ at the plasmonic resonant frequency with illumination power of $9.2\,{\rm{nW}}$ at 6.5 K. The responsivity of the device can be further increased by operating it at even lower temperatures and weaker illumination. From the above equation for ${R}_{{Vc}}$, it is expected that lower temperature leads to larger responsivity, as proven by our experimental data shown in Fig. 2d. Additionally, there exists a minimum power that can trigger phase slip events and lead to superconducting transition. Our plasmonic structure enhances the device’s efficiency in harvesting the target infrared light, thereby lowering threshold power. In our case (See Supplementary Note 7), the measured minimum power that can trigger the phase slip events is $23\,{\rm{\mu }}{\rm{W}}\,{{\rm{cm}}}^{-2}$ (2.3 nW in sensor) at 6.5 K, corresponding to a highest responsivity of $7.4\times {10}^{8}\,{\rm{V}}{{\rm{W}}}^{-1}$. Considering the noise characteristics, the thermal Johnson noise of our device is evaluated to be on the order of $1.7\times {10}^{-9}\,{\rm{V}}{{\rm{Hz}}}^{-1/2}$ and the shot noise, $6.27\times {10}^{-8}\,{\rm{V}}\,{{\rm{Hz}}}^{-1/2}$ at the typical current of 200 $\mu A$ which is found to be dominant in the high frequency range $\ge 100\,{\rm{kHz}}$ (At lower frequencies, 1/f noise shows up). As a result, the minimum noise equivalent power (NEP) of our device is $8.5\times {10}^{-17}\,{\rm{W}}\,{{\rm{Hz}}}^{-1/2}$, and the specific detectivity is ${D}^{* }=1.2\times {10}^{14}\,{\rm{Jone}}$, which is comparable to reported works^42^ (see also Supplementary Table S1 in supplementary information). In addition, we mention that this noise level does not affect the memlogic operation of our device because discrete current or light intensities are adopted, which is intrinsically resistant to the noise, as evident by the excellent signal-to-noise ratio in measured time-trace of the resistance-state (e.g., Fig. 2b).

**Fig. 3:**
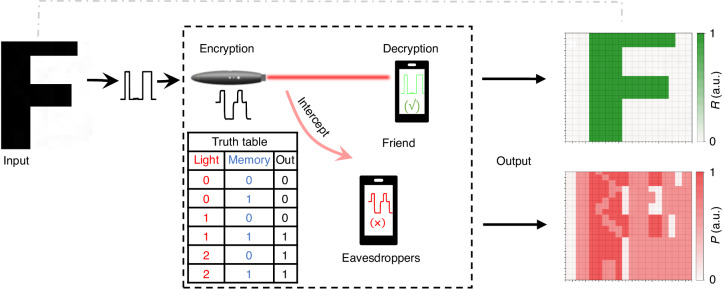
Optical bistability applied in encryption. A binary picture “F” is firstly changed to one-dimensional array ASCII, and then is encrypted by algorithm basing on devices’ truth table. Next the laser will transmit the encrypted information with three types of intensity of light power to the sensor. The eavesdropper intercepts the information with the traditional sensor (such as power meter) and obtains a false picture with three different types of intensity, because the light 1 cannot be simply regarded as logic 0 or 1. Only the friend obtains the right picture with our memlogic infrared sensor, which automatically decrypted the information thanks to its memlogic functions

The unique optical controlling bistability characteristic can be applied in, e.g., secure optical communication in invisible spectrum range. Figure 3 shows a schematic diagram and measured data. A binary picture “F” is first converted to a one-dimensional array ASCII code and then encrypted using an algorithm based on the device’s truth table (inset in Fig. 3). The laser then transmits the encrypted information to the sensor. If an eavesdropper intercepts the information on this process with the traditional sensor (such as power meter), he will obtain one-dimensional array with three different types of intensity. The measured data is showed in right bottom of Fig. 3. In the traditional way, one may set a threshold value, and the signal can be discretized to either 1 or otherwise 0. This is completely wrong for decrypting our information because the middling intensity (light 1) cannot be simply regarded as 0 or 1; instead it depends on the previous state. Our memlogic sensor, however, automatically decrypts the information correctly (right top of Fig. 3), thanks to its memlogic functions.

### Reconfigurable memlogic circuit

**Fig. 4:**
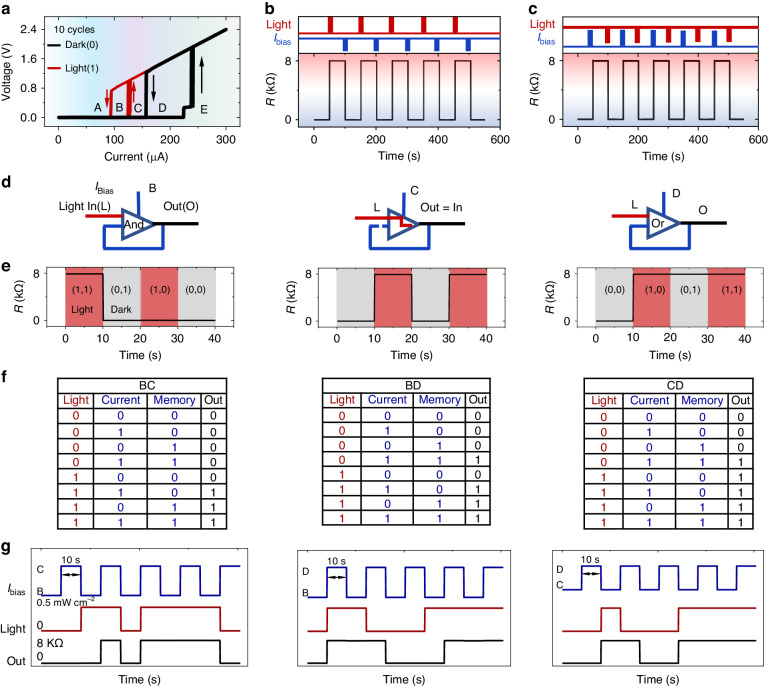
Reconfigurable memlogic characteristics. **a** Ten cycles sweeping I-V hysteretic curves of memlogic sensor with (solid red line) and without (solid black line) light illumination ($0.5\,{\rm{mW}}\,{{\rm{cm}}}^{-2}$) at $6.3\,{\rm{K}}$. **b** Write to HRS by optical pulse ($0.5\,{\rm{mW}}\,{{\rm{cm}}}^{-2}$, duration of 0.3 s), and ease to LRS by low electrical pulse ($1\,{\rm{\mu }}{\rm{A}}$, duration of $0.3\,{\rm{s}}$), reading at $180\,{\rm{\mu }}{\rm{A}}$ with light off. **c** High electrical pulse ($400\,{\rm{\mu }}{\rm{A}}$, duration of $0.3\,{\rm{s}}$) is used to switch the LRS to HRS with light illumination ($0.5\,{\rm{mW}}\,{{\rm{cm}}}^{-2}$). The HRS is switched to LRS by turning off light with duration of $0.3\,{\rm{s}}$. Reading operated at current of $110\,{\rm{\mu }}{\rm{A}}$. **d** schematic of reconfigurable memlogic device and circuit design. The device can used as AND, light signal follower and OR, respectively. **e** Output of AND, follower and OR operation. **f** Truth table for square wave bias current operation. **g** Testing data of square wave bias current operation

In addition to the pure electrical/optical switching characteristic, we next program the device using cooperatively optical stimulations and electrical pulses. Ten repeated cycle hysteretic IV curves with illumination of $0.5\,{\rm{mW}}\,{{\rm{cm}}}^{-2}$ (red curves) and without intentional light illumination (black curves) are shown in Fig. 4a, respectively, demonstrating its excellent repeatability and robustness. We defined operating bias current zone as A, B, C, D and E for ${{\rm{I}}}_{{bias}} < 93\,{\rm{\mu }}{\rm{A}}$, $93\,{\rm{\mu }}{\rm{A}}$ $< {{\rm{I}}}_{{bias}} < 124.2\,{\rm{\mu }}{\rm{A}}$, $124.2\,{\rm{\mu }}{\rm{A}}$ $< {{\rm{I}}}_{{bias}} < 157.2\,{\rm{\mu }}{\rm{A}}$, $157.2\,{\rm{\mu }}{\rm{A}}$ $< {{\rm{I}}}_{{bias}} < 223.2\,{\rm{\mu }}{\rm{A}}$ and $223.2\,{\rm{\mu }}{\rm{A}}$ $< {{\rm{I}}}_{{bias}}$, respectively. In one instance, the device initially operated at LRS with a bias current of $180\,{\rm{\mu }}{\rm{A}}$ (D zone), it is switched to HRS under the light illumination with a wavelength of 12.2 ${\rm{\mu }}{\rm{m}}$, a power density of $0.5\,{\rm{mW}}\,{{\rm{cm}}}^{-2}$, and a duration of 0.3 s. The device retained the HRS even after the light is removed. The reset process is initiated by an electrical pulse ($1\,{\rm{\mu }}{\rm{A}}$, 0.3 s), shown in Fig. 4b. In another case, the device initially operated at LRS with a bias current of $110\,{\rm{\mu }}{\rm{A}}$ (B zone) and illumination with a power density of $0.5\,{\rm{mW}}\,{{\rm{cm}}}^{-2}$. The set process is induced by a high electrical pulse ($400\,{\rm{\mu }}{\rm{A}}$, 0.3 s), The reset occurred after sudden cessation of the light (0.3 s), as illustrated in Fig. 4c. These cooperative optical stimulation and electrical pulse-based set/reset processes are highly repeatable and robust (Fig. 4b, c).

When the devices are operated at different zone of constant bias current, it performed different functions. This non-volatile optoelectronic switching characteristics of our sensor can be further applied in reconfigurable sequential memlogic circuits, as shown schematically in Fig. 4d. This memlogic circuit consists of two inputs (light signal and memory state), one electrical selector and one output. When operated at current bias in B, C and D zones, this memlogic device performs the “AND”, “light signal follower” and “OR” functions, respectively. For instance, during “AND” operation, only the combination of light on (1) and memory state HRS (1) results in an HRS output. The initial HRS can be obtained by a high electrical pulse (400 μA, 0.1 s). When we operate the sensor at a bias current in zone B, the output follows the light on/off. At the bias current in zone D, the output is LRS (0) only when the light is off (0) and LRS memory state. The testing data is shown in Fig. 4e. A more complex logic function can be generated by applying a square wave current between the B, C, and D zones. In this way, the output depends not only on the light signal input and memory state but also on the biased current state. The truth table and testing data for sequential logic are shown in Fig. 4f and g, respectively. The bias current is established 100 ms before the optical input signal during the test. Achieving these sequential logic functions in a single device eliminates the need for integration of several devices that is usually required with conventional CMOS devices. Therefore, the optical and electrical-set/reset operations allow the present single device to be a reconfigurable logic element that may be used to switch between different algorithms to efficiently reduce the circuit complexity and increase the effective integrating density of the processor chip. All the above sequential logic functions being achieved in single device level may provide promising applications in a reconfigurable superconducting logic circuit, superconducting computer and intelligent remote sensing and communications, as we exemplify in the following.

**Fig. 5:**
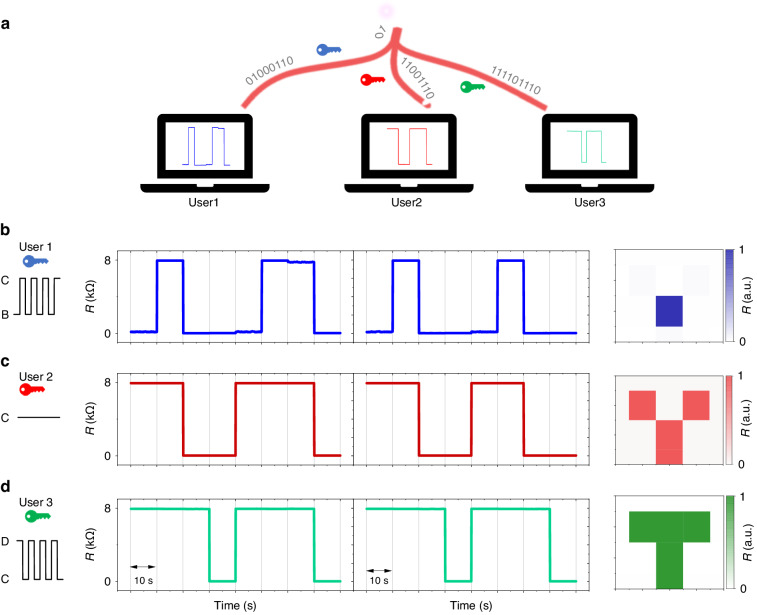
Information encryption for implementation of reconfigurable memlogic sensor. **a** Schematic diagram of decoding information with different key. **b**–**d** showing the information obtained by User1, User 2 and User 3 with blue key (square wave current bias between B and C zone), red key (constant current bias at C), and green key (square wave current bias between C and D zone, respectively. The B, C, and D are indicated at Fig. 4a

Figure 5 demonstrates the implementation of optically encrypted information transmission using our sensor’s reconfigurable memlogic capabilities. We obtained diverse information from the same optical information by operating the sensor at different bias currents corresponding to distinct keys, as shown Fig. 5b–d. The square wave bias current between B and C, the constant bias current C and the square wave bias current between C and D represent the blue, red and green keys, respectively. Only the correct key will allow the user to access the correct information. The encrypted method demonstrated in Fig. 3 ensures security during transmission. Correct information can only be accessed using the appropriate key, providing an additional layer of security in terminal equipment, as illustrated in Fig. 5. In our demonstrated cryptography, it is crucially important that the receiver needs the correct key (electrical pulse sequence) in order to decrypt correctly corresponding information. Suppose the correct information to be transmitted is “T”, only electrical pulse alternating between C [135 μA] and D [180 μA] works as the correct key and gives the correct decryption result (see Fig. 5d). Therefore, even if eavesdroppers possess the ability to duplicate our device, decryption with wrong keys will give wrong information and the eavesdropper will be deceived (Fig. 5b, c). Compared to other visible light encrypted technology^43^, the invisible infrared light implemented here in encrypted information transmission brings more spectral freedom and may be more beneficial for confidentiality because the correct information is prevented from being intercepted by other (conventional) infrared sensors or similar sensors without the correct key.

**Fig. 6:**
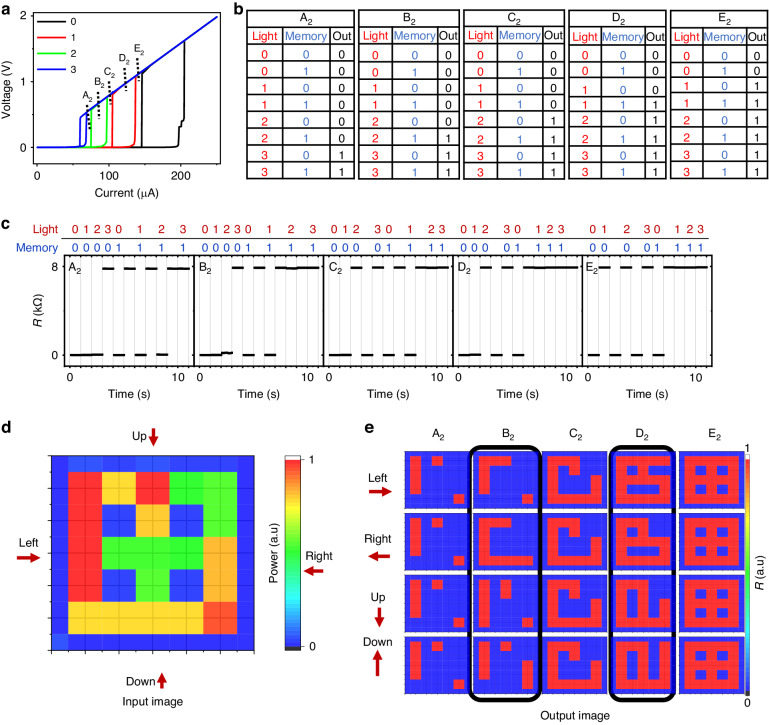
Controlled by multiple state of light and bias current. **a** I-V curve for device under light illumination with intensity of $0,1$ ($0.19\,{\rm{mW}}\,{{\rm{cm}}}^{-2}$), 2 ($0.38\,{\rm{mW}}\,{{\rm{cm}}}^{-2}$) and $3(0.65\,{\rm{mW}}\,{{\rm{cm}}}^{-2})$ at $6.5\,{\rm{K}}$. The zone of A_2_, B_2_, C_2_, D_2_ and E_2_ represent bias current $68\,{\rm{\mu }}{\rm{A}} < {I}_{{Bias}} < 75\,{\rm{\mu }}{\rm{A}}$, $75{\rm{\mu }}{\rm{A}} < {I}_{{Bias}} < 96\,{\rm{\mu }}{\rm{A}}$, $96\,{\rm{\mu }}{\rm{A}} < {I}_{{Bias}} < 103\,{\rm{\mu }}{\rm{A}}$, $103\,{\rm{\mu }}{\rm{A}} < {I}_{{Bias}} < 135\,{\rm{\mu }}{\rm{A}}$ and $135\,{\rm{\mu }}{\rm{A}} < {I}_{{Bias}} < 144\,{\rm{\mu }}{\rm{A}}$, respectively. **b** Truth table for device under the operation of A_2_, B_2_, C_2_, D_2_ and E_2_, respectively. **c** Output of resistance under the operation of A_2_, B_2_, C_2_, D_2_ and E_2_. **d** The input light image with four states of light intensity. **e** The different output image of device under the different reading current and different direction of light signal transmission

We conducted further investigations into the transmission of multiple states of light intensity. In Fig. 6a, various IV curves are displayed under light illumination with four different power densities (0 for light off, and 1–3 corresponding to 0.19, 0.37, and 0.65 ${\rm{mW}}\,{{\rm{cm}}}^{-2}$). Under illumination by each light intensity state, the device operating at the bias current in A_2_, B_2_, C_2_, D_2_, and E_2_ zones will yield five different truth tables, respectively, as illustrated in Fig. 6b. Figure 6c presents the sequential logic testing data. These four light intensity states are compressed into the HRS and LRS on the devices and can be distinguished by operating at different bias currents. When operating at the bias current in C_2,_ it functions as an analog to digital converter (ADC) converting an analog light signal ($> 0.37\,{\rm{mW}}\,{{\rm{cm}}}^{-2}$) to digital 1 (HRS) and a light signal ($< 0.19\,{\rm{mW}}\,{{\rm{cm}}}^{-2}$) to digital 0 (LRS). What’s more, it can generate more information due to memlogic characteristics. Theoretically, if there are N kinds of light intensity states, and the regions included in the corresponding IV hysteresis curve do not overlap, then 2N-3 different truth tables will be generated under operation at certain bias current. Figure 6b and Supplementary Fig. S7 showed 4 and 3 light intensity states that corresponded to 5 and 3 different truth tables, respectively. Figure 6d displays the image encoded by 4 kinds of light intensity. This image is arranged in a one-dimensional array from various directions and then sent to the sensor. The sensor yields five different images when operated at five different current bias (A_2_, B_2_, C_2_, D_2_, and E_2_). Additionally, the memlogic feature of the device in regions B_2_ and D_2_ results in a unique image under the illumination of different light sequences in various directions (Up, Down, Right, Left), as shown in Fig. 6e. This method of multi-value optical information compression and restoration also provides a new way for the encrypted transmission of information. Moreover, suppose the device is made into an array and works in different bias current regions. In that case, the parallel transmission of optical information can be realized, as shown in Supplementary Fig. S8.

## Discussion

As mentioned before, our memlogic superconducting sensor is distinguished from existing superconducting photon detectors such as SNSPDs and TESs. Our sensor is operated with bistable states with constant current positive feedback control mode. The state can be switched by electrical pulse and/or optical pulse and can be memorized for a long time. We also present its reconfigurable memlogic function. However, the bistable states are typically not favorable for conventional superconducting photodetectors. For instance, the SNSPD is operated at superconducting state with constant current negative feedback control mode. It is optimized such that the weak light pulse can be detected with single-photon sensitivity, meanwhile without the memlogic function. The TES is operated near-constant voltage bias to stabilize the device in the superconducting transition edge via negative electrothermal feedback. It’s very sensitive to the temperature change and without the memlogic function. Compared with other current-induced phase change or Joule-heating-based superconducting devices, our memlogic sensor also deviates from the cryotron^44^, nanocryotron (ntron)^45^ and htron^46^. They use an input gate current or adjacent heater to induce phase/resistivity changes in the channel. Although the cryotron has the function of memory and logic computing, its function usually is fixed after it is manufactured. Besides, no optical sensitivities have been attempted. Distinctively, our sensor has the reconfigurable memlogic function and can be encoded by optical pulse, paving the way to optical input for logic computing. See Supplementary Note 12 and Supplementary Table S1 for the more detailed comparison of our device with other existing technologies. We mention that, inheriting from the simple structure of other Joule-based superconducting devices, our device is possible to be constructed into array which is highly favorable for applications like infrared imaging, on-chip spectrometers, data parallel transmission using different wavelengths, on-chip image processing, and recognition (see, e.g., Supplementary Note 10 and Note 11), among others.

To this end, the power consumption assessment is crucially important. To evaluate the power consumption of our devices, it is noteworthy to highlight that the LRS is dissipationless due to the flowing supercurrent. This is reminiscent of the off state of a semiconductor transistor in low dissipation state, however, due to absence of electrical current. The current power consumption of our device stands at approximately ~600 μW, translating to a thermal power density of around ~60 nW μm^−2^, when operated at HRS. As a reference, it’s noteworthy that the power density of typical commercial semiconductor chips at room temperature, such as a central processing unit (CPU), hovers around ~400 ${\rm{nW}}\,{{\rm{\mu }}{\rm{m}}}^{-2}$ ^47^ and phase-change memory (PCM)^48^ ~ 5 ×10^7^ nW μm^−2^. Compared to other reported superconducting logic circuit devices, our device’s power density appears also reasonable since ntron consumes a typical power density of ~80 ${\rm{nW}}\,{{\rm{\mu }}{\rm{m}}}^{-2}$ ^49^ and htron, ~10,000 nW μm^−2^ ^46^. With the same power density level (~60 nW μm^−2^), our device can be scaled down to~10 ×10 μm² and a thermal power consumption of ~6 μW can be expected. Noting the typical cooling power (~5 W@ 6.5 K) provided by our cryogenic system, the array size can reach ~100,000 pixels concurrently running. This significantly exceeds the array size of TES bolometer devices, typically comprising only a few thousand pixels^50^, and aligns with the maximum pixel number of very recent SNSPDs (~400,000 pixels)^51^. Further optimization of the power budget may be achieved by improvements in thermal isolation or reduction in the critical current density and resistivity of each pixel (e.g., leveraging membrane support instead of a bulky substrate). Alternatively, increasing the operational temperature (*T*_*c*_ of the superconducting material, such as YBCO) would substantially boost the cooling power, thereby expanding the total pixel count of the array device.

In conclusion, our study showcases a memlogic Nb sensor integrating the function of infrared sensitivity, memory and reconfigurable logic computing. Based on in-sensor computing architectures, we introduced an optical encryption transmission technique. These functions demonstrated in our work can also be achieved with high temperature superconductor, since the bistable effect is common for superconductors. Our work opens a new avenue for superconducting in-sensor computing and intelligent infrared sensing and communication.

## Materials and methods

### Sample and device fabrication

The memlogic device contained three layers 60 nm/280 nm/100 nm Nb/Si/Nb. The bottom metal mirror is patterned using UV lithography and 100 nm Nb is deposited on thermally oxidized Si wafers using a magnetron sputtering machine. The base pressure during deposition is less than $3\times {10}^{-7}$ torr, with a deposition rate of ~3.7 ${\rm{\mathring{\rm A} }}$, DC power of 200 W, and Ar gas pressure of 2 mtorr. The bottom layer’s area is $400\,{\rm{\mu }}{\rm{m}}\times 400\,{\rm{\mu }}{\rm{m}}$, which is smaller than that of wafers and larger than that of the top layer. A 280 nm continuous Si film is then deposited on the bottom layer and thermally oxidized Si wafers by the same magnetron sputtering machine with a base pressure of less than $3\times {10}^{-7}$ torr (at a rate of ~0.8 ${\rm{\mathring{\rm A} }}$ with an RF power of 150 W and Ar gas pressure of 2 mtorr). The top layer of 60 nm thick Nb continuous film is deposited on Si film and a 2 nm Si film is then deposited on Nb film to prevent oxidation. The top layer is patterned using e-beam lithography (EBL) with negative electron beam photoresist AR7520, and the exposed area is then etched by Reactive Ion Etching (RIE) with CF_4_ gas.

### Optical spectrum measurements

The optical reflectance spectrum(*R*) of the device is measured by FTIR system (Burke V80, 15X objective) at room temperature with the normalized background of the reflectance spectrum of the gold reflector. The absorption spectrum(*A*) is determined as *A* = *1-R* considering that the bottom layer of the 100 nm Nb reflector blocks light transmission (T = 0). In addition, a polarizer is used to obtain the polarized light, with the electrical filed being almost perpendicular to short axis of Nb wire.

### Optical and electrical measurements

All electrical measurements are carried out in variable temperature cryostat with $\pm 3\,{\rm{mK}}$ stability (PHYSIKE: Qcryo Scryo-S-300). I-V hysteresis, endurance and retention test are measured by home-built LabVIEW programs with constant current source (Keithley 2450) and voltmeter (Keithley DM6500). The duration of each bias current point is 100 ms, when measuring the I-V hysteresis curve. The time response of electric pulse switching between the HRS and LRS is measured by connecting a series 10 kΩ resistance to a square wave voltage that ranged from 0.1 V and 5 V. An oscilloscope (Tektronix: MDO3014) recorded this time response, as shown in Supplementary Fig. S3c. The square wave is generated by an arbitrary function generator. (Tektronix: AFG 31000).

The optical characteristic of the memlogic device is measured by a tunable quantum cascade laser (MOLECULAR VISTA: LASB0000-C-0006) with a repetition of 0.5 MHz and duration of 100 ns. Two polarizers are placed in QCL box. The power density of light is tunable by adjusting the angle of one of the polarizers and is detected by a power meter (Thorlabs: S401C). The responsivity (*R*_*v*_) is defined as ${R}_{V}=\frac{{{Voltage}}_{{light\; on}}-{{Voltage}}_{{dark}}}{{Power}}$. As shown in Fig. 2d, we measure the responsivity by sweeping current bias at each fixed temperature point with light on and light off. At a certain temperature, the max responsivity is derived from the voltage of the normal state induced by light minus the voltage of superconducting state at dark (almost zero). The time response of light switching the HRS and LRS of device is recorded by oscilloscope at 6.5 K with chopper modulated the QCL on/off, as shown in Supplementary Fig. S4.

### Memlogic characteristic and application

The memlogic characteristic and applications are measured by home-built Labview programs. During the sequential logic test, as shown in Figs. 4g and 5b–d, we programmed the polarizing rotary motor and source meter to generate Optical and electrical synchronizing signals.

### Supplementary information

Supplementary Information for Reconfigurable memlogic long wave infrared sensing with superconductors
