# Supplementary material for: Reconfigurable memlogic long wave infrared sensing with superconductors
Source: Light Sci Appl. 2024 Apr 26;13:97. doi: 10.1038/s41377-024-01424-2 (PMC11053096; doi:10.1038/s41377-024-01424-2)
Supplement: Supplementary file 1 — Supplementary Information for Reconfigurable memlogic long wave infrared sensing with superconductors [file 41377_2024_1424_MOESM1_ESM.pdf]

# Supplementary Information for

# Reconfigurable memlogic long wave infrared sensing with superconductors

Bingxin Chen[1], Huanyi Xue[1], Hong Pan[1], Liping Zhu[1], Xiaomi Yan[2], Jingzhe Wang[2]
Yanru Song[2,*] and Zhenghua An[1,3,4,5,*]

[1]State Key Laboratory of Surface Physics and Institute for Nanoelectronic Devices and Quantum Computing, Department of Physics, Fudan University, Shanghai, China

[2]ShanghaiTech Quantum Device Lab, ShanghaiTech University, Shanghai 201210, China

[3]Shanghai Qi Zhi Institute, 41th Floor, AI Tower, No. 701 Yunjin Road, Xuhui District, Shanghai, 200232, China

[4]Yiwu Research Institute of Fudan University, Chengbei Road, Yiwu City, 322000 Zhejiang, China

[5]Zhangjiang Fudan International Innovation Center, Fudan University, Shanghai 201210, China

*E-mail: songyr@shanghaitech.edu.cn (Y.S.), anzhenghua@fudan.edu.cn (Z.A.)

## Supplementary information

Content

1. Additional data of electrical characterizations
2. Simulation of electrical field distribution
3. Tunable optical properties of metamaterials
4. Time response of optical switching characteristic
5. Temperature dependence of hysteresis and mechanism
6. The simulation of heat transfer
7. Responsivity of sensor
8. The memlogic characteristic with three intensities of light
9. Schematic diagram of parallel information transmission in memlogic arrays
10. Schematic diagram of ANN memlogic array
11. Potential application of memlogic arrays
12. Comparison with others similar devices
13. Reference

## 1. Additional data of electrical characterizations

The critical temperature of the device is measured with a small constant bias current of 1 μA and a four-probe method, revealing that the superconducting state is completely disrupted at approximately 7.3 K, as displayed in Fig. S1a.

To assess the reliability and endurance of our memlogic devices, we conducted an endurance experiment wherein an electrical pulse (400 μA and 0.1 s duration) is applied during the program process, while a smaller electrical pulse (1 μA and 0.1 s duration) is involved for reset process, and HRS/LRS are read under a bias current of 200 μA. The on/off ratio reached up to $10^3$ over $10^6$ cycles, as illustrated in Fig. S1b. At lower temperatures, theoretically, this on/off ratio can be infinite because of zero resistance in the superconducting state. The HRS/LRS remained steady as a function of the cycle number, demonstrating the robustness of our memlogic sensor's memory.

We also assessed the time response of switching the HRS and LRS. A series of 10 KΩ resistance is utilized to generate a small constant bias current within a low voltage supply. The program process and reset process, which lasted for 10 μs, operated with high voltage of 5 V and low voltage of 0.1 V respectively, as produced by arbitrary function generator. Under high electrical pulse, the device's voltage attained a plateau within 2.2 μs whereas it dropped back to zero under low electrical pulse in just 1.9 μs, as shown in Fig. S1c.

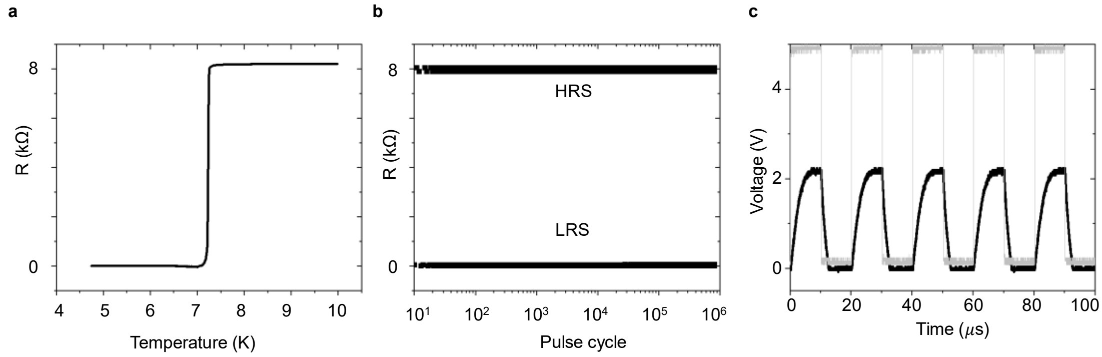


**Figure S1 Additional data of electrical characterizations. a** The resistance-temperature curve show the critical temperature is about 7.3 K. **b** Endurance characteristics of memlogic sensor, showing no error during $10^6$ pulse cycle. The HRS is programmed by high current pulse (400 μA, duration of 0.1 s), and the reset process is initiated by a low current pulse (1 μA, duration of 0.1 s), read by current 200 μA. **c** Time response of switching the HRS and LRS. The grey square wave voltage is generated by arbitrary function generator with low level 0.1 V and high level 5 V.

## 2. Simulation of electrical field distribution

The finite-difference time-domain (FDTD) method is implemented for the simulation in which we set the permittivity of Si $\varepsilon = 11.9$, and the permeability constant $\mu = 1$. The permittivity of Nb is sourced from reference [1]. The electric field distribution of x-y and x-z cross section of Nb-Si-Nb unit cell are shown in Fig. S2. Localized surface plasmons are excited along the short Nb wire axis under perpendicular polarized light (along the z axis). As seen in Fig. S2, the electric field is primarily confined to the top Nb wire with an approximately 10-fold enhancement. The maximum incident

electric field is $\sim 1.2 \times 10^{7} \mathrm{Vm}^{-1}$.

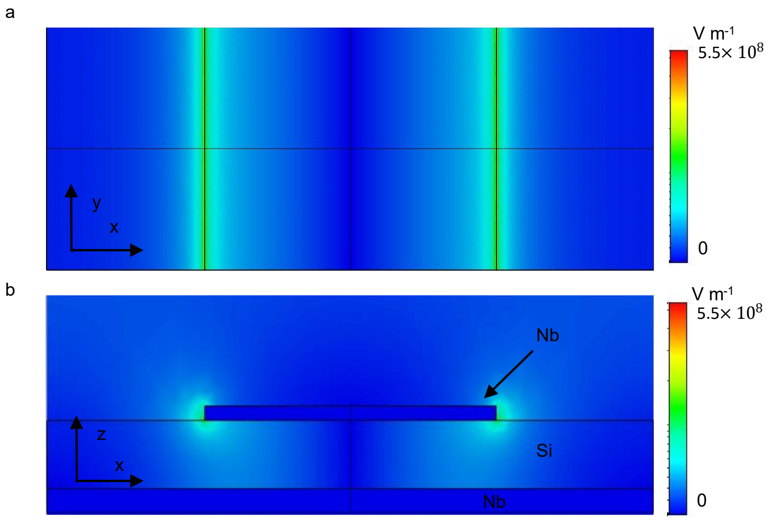


**Figure S2 Simulation of electric field distribution**. **a** The x-y plane electric field distribution. **b** The z-x plane electric field distribution.

## 3. Tunable optical properties of metamaterials

The resonant wavelength of metamaterial is easily tunable by adjusting the width of Nb wire, as demonstrated in Fig. S3a and S3b while maintaining fixed Nb and Si thickness and period. The width of Nb wire is tuned form 0.8 μm to 1.3 μm. The resulting absorption spectrum of each varied width is displayed in Fig. S3a. A trend line between the wavelength and width is plotted from extracted resonant wavelengths in Fig. S3a, as presented in Fig. S3b. As discussed in the main text, there exists a linear dependence between Nb wire width and resonant wavelength (Fig. S3b).

Metamaterial's absorption intensity can be adjusted by changing the period of the material, as depicted in Fig. S3c. The resonant absorption intensity initially increases with increased period but declines following peak absorption at a period of 2 μm.

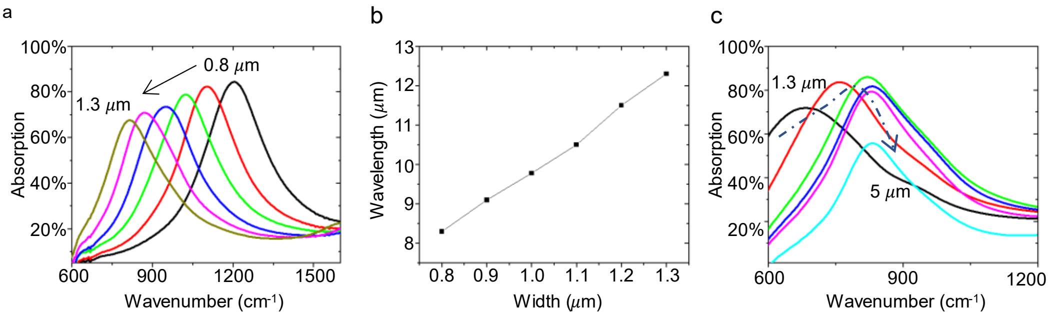


**Figure S3 Tunable optical properties of metamterials a** The absorption spectrum of different width of metamaterial. **b** The resonant of wavelength dependent on width of metamaterial. **c** The absorption spectrum of different period of metamaterial.

## 4. Time response of optical switching characteristic

We measured the optical switching characteristic's time response by recording voltage time traces, as depicted in Fig. S4, using an oscilloscope at 6.5 K, constant

voltage source (9 V), and a 100 kΩ resistance in series. The infrared light (12.2 μm, 0.66 mW $cm^{-2}$) is modulated by a chopper for on-off signal processing. Writing and erasing via light resulted in response times of 5.4 ms and 4.5 ms respectively. This time response of optical switching is slower than electrical switching. The response time of switching main contain the thermal relaxation and electrical recovery time $\tau = L/R$, where the $L$ is kinetic inductance of Nb wire, $R$ is the normal state resistance. The light radiation not only heats up the Nb wire but also the substrate, potentially prolonging the thermal relaxation time compared to electrical switching. Operational speed can be further improved by reducing the device's size, thereby decreasing resistance and parasitic inductance/capacitance. Additionally, drawing from the concept of the Joule heat superconducting device[2], a novel approach could enhance the speed to the thermal relaxation time (~ns). This involves replacing the metallic electrical heater in the htron with an optical antenna, commonly used to harvest light and generate local hotspots[3-5].

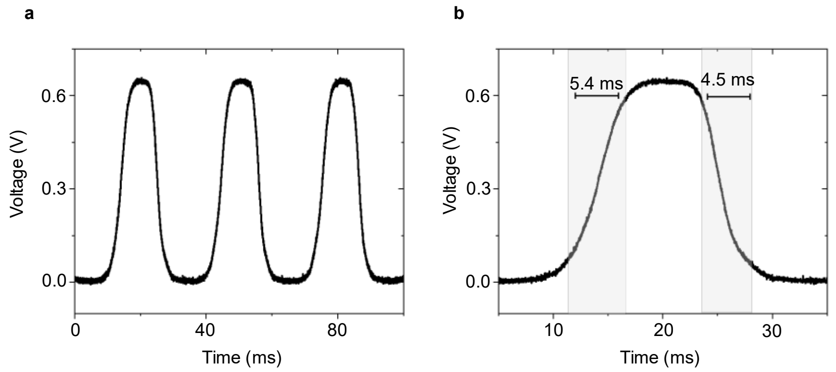


**Figure S4 Time response of optical switching characteristic**. **a** It is recorded by oscilloscope at 6.5 K with constant voltage series with 100 kΩ resistance. The light on-ff is modulated by chopper. **b** The response time of writing and erasing by light is 5.4 ms and 4.5 ms, respectively

## 5. Temperature and light power dependence of hysteresis and mechanism

We begin by considering a simple one-dimensional model appropriate to bridges which are much longer than the thermal healing length $\eta$. The typical thermal healing length is of order 5 μm. Heat generated in localized dissipative regions in such bridges is transferred in two ways: by thermal conduction within the film and by surface heat transfer across the temperature discontinuity which develops at the boundary with the substrate (and with the helium bath, if present). Because the dimensions of the microbridges are very small compared to the dimensions of the substrate, the substrate may be assumed to be essentially at the ambient temperature $T_b$. We assume a bridge of length $L$, width $W$, and thickness $d$ with a normal region of length $2x_N$ and resistivity $\rho$, symmetrically centered in the bridge. The temperature distribution $T(x)$ along the bridge must satisfy the heat-flow equations[6]:

$$-K_N \frac{d^2T}{dx^2} + \frac{\alpha}{d}(T - T_b) = \left(\frac{I}{Wd}\right)^2 \rho \quad (|x| < x_N) \quad (1)$$

$$-K_S \frac{d^2T}{dx^2} + \frac{\alpha}{d}(T - T_b) = 0 \quad (|x| > x_N) \quad (2)$$

where $K_N$ and $K_S$ are the thermal conductivities of the wire in the normal and superconducting

states, respectively. $\alpha$ is the total heat transfer coefficient per unit area of film, and $I$ is the current flowing through the bridge. The temperature at $\pm x_N$ is assumed to be $T_C$. The temperature profile along the wire, $T(x)$, is solved for assuming that the heat flow at $\pm x_N$ is continuous, and $T \approx T_b$ as $x \rightarrow \pm\infty$. By solving for a range of $x_N$, we can trace out a current-voltage (IV) curve that contains a distinct region of near-constant current for a range of voltages. This current is the hotspot current, $I_r$.

When the hotspot is sufficiently long, in our case, the entire wire is in normal state with constant temperature $T_c$ due to Joule heating, such that $\frac{d^2T}{dx^2} \approx 0$, allowing us to drop the first term on the left-hand side of (1):

$$\frac{\alpha}{d}(T_c - T_b) = \left(\frac{I_r}{Wd}\right)^2 \rho \qquad (3)$$

Thus, we can get the retrapping current $I_r = (\alpha W^2 T_c d/\rho)^{1/2}(1 - T/T_C)^{1/2}$ without illumination of light. This is the threshold current that can keep the entire wire normal state due to Joule heating. When the bias current smaller than $I_r$, the wire will be superconducting state.

The above equation (3) can also be rewritten as[7]:

$$\alpha W^2(T_c - T_b) = {I_r}^2 R_n \qquad (4)$$

Where the $R_n$ is the sheet resistance in the normal state.

Next, when we illuminate the light on wire, another heating source caused by light power needed to be consider into the heat-flow equations. Thus, the equation (4) can be modified as:

$$\alpha W^2(T_c - T_b) = {I_r}^2 R_n + \beta SP \qquad (5)$$

Where $\beta$ is an adjustable parameter, P is incident light power density, $S$ is the area of sensor. We can get the modified retrapping current $I_r(P)^2 = \alpha W^2(T_c - T_b)/R_n - \frac{\beta S}{R_n} \cdot P$.

## 6. The simulation of heat transfer

We use the Multiphysics software to simulated the thermal balance physics to further verify our hot spot model. In our model, the substrate is assumed to be essentially at the ambient temperature Tb, due to the dimensions of the substrate is very lager compared to the area of nanowire sensor. The Joule heating is the only heating source. The Joule heating would be transfer into the substrate. The interface of thermal conductivity $\alpha = 5.86\ \mathrm{W\ cm^{-2}\ K^{-1}}$. The geometry of Nb nanowire is set the same as the experiment data, width $W$=1.3 μm, length $L = 100$ μm and period $P = 2.5$ μm. The resistivity of Nb wire is also the same as measurement data $\rho = 1.56 \times 10^{-7}\Omega\mathrm{m}$. When we set different input current $I_{bias}$ and different ambient temperature $T_b$, it would generate new thermal balance temperature $T$ alone the nanowire sensor. When the temperature of nanowire larger than the critical temperature $T_C$, the nanowire sensor is in normal state with normal resistance, otherwise the nanowire sensor is in superconducting state with zero resistance. When we set the temperature of nanowire equal to $T_C$, we can obtain a serials of ambient temperature $T_b$ and input current $I$. This simulation data is quite agreed with our experimental data and theory (Fig. S5).

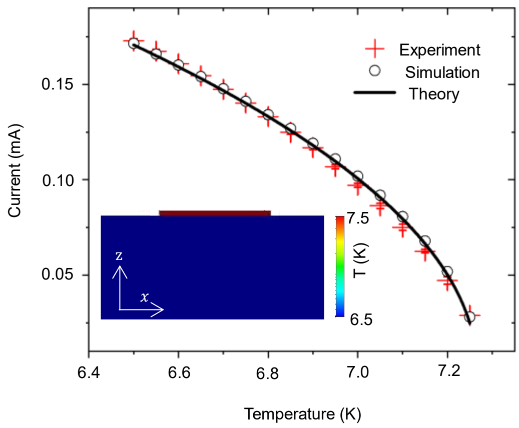


**Figure S5 The retrapping current vs temperature.** Inset: the simulation of temperature distribution of cross section of one period of Nb wire and substrate. The temperature of substrate $T_b$=6.5 K, the bias current $I_{bias}$=0.2 mA.

## 7. The responsivity of our sensor

$I_c$ is derived from IV curves under varying light intensity, and averaged ten times, plotted against light power in Fig. S6, with power averaged eighty times. There is $I_c$ plateau when the light power is small enough. Because there is no enough energy to destroy enough quantities of Cooper pairs and to trigger the superconducting transition. After exceeding a light intensity of $23\ \mu\mathrm{W\ cm}^{-2}$, $I_c$ significantly decreased; thus the maximum responsivity at 6.5 K, is $R_{Vc} = \frac{I_c(P)R}{P} = 7.4 \times 10^8\ \mathrm{VW}^{-1}$.

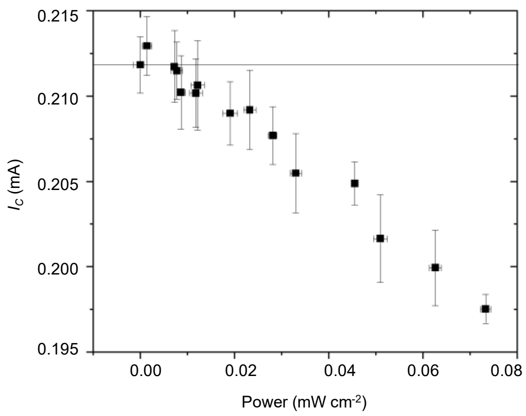


**Figure S6 The critical current suppressed by intensity of light**. When the light intensity exceeds a certain value, the current decreases with the increase of the light intensity.

## 8. The memlogic characteristic with three intensities of light

We measure the IV curves under light illumination with three different power densities of 0 (light off), 1(0.4 mW cm$^{-2}$), and 2(0.65 mW cm$^{-2}$) at 6.5 K, as shown in

Fig. S7a. Different output states are obtained when operating at various bias current zones despite receiving the same light input. In the case of three intensity of light, we can define three operating bias current zone, corresponding to A, B, C in Fig. S7a. The three different output state and logic truth table is shown in Fig. S7b. When an encrypted letter “B” image with three different intensities of light transmit to sensor by laser, we can decode to a letter “L”, “E” or “B” through operating the sensor at different bias current A, B or C, respectively. (Fig. S7)

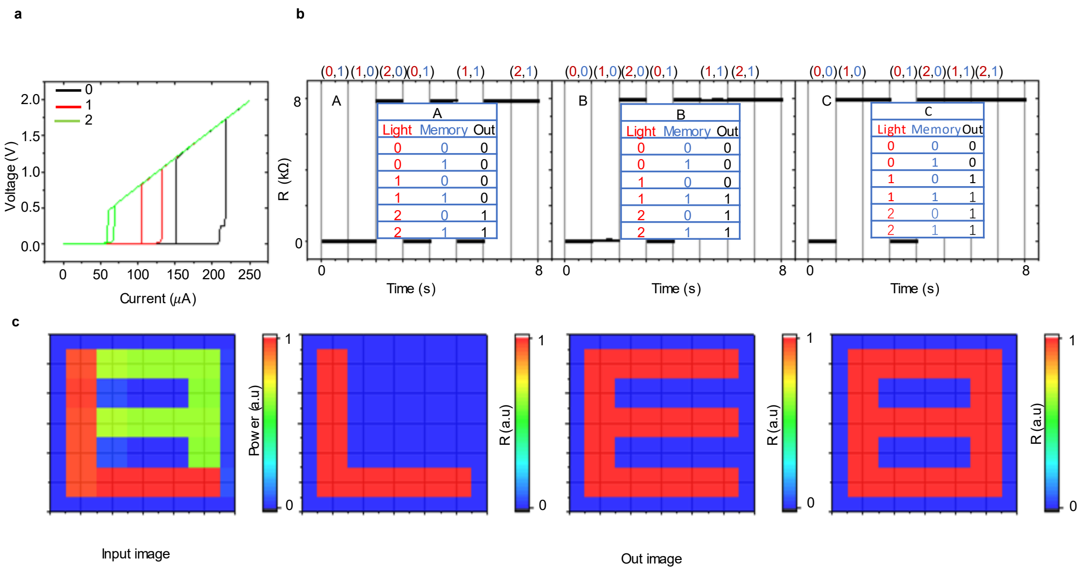


**Figure S7 The memlogic characteristic with three intensities of light**. **a** I-V curve for device under light illumination with intensity of 0, 1 (0.4 mW cm$^{-2}$), and 2 (0.65 mW cm$^{-2}$) at 6.5 K. **b** Truth table for device under the operation of bias current A, B, and C, respectively. **c** Different image is obtained by the operation of bias current A, B, and C.

## 9. Schematic diagram of parallel information transmission in memlogic arrays

We use a memlogic sensor to simulate the parallel information transmission. The schematic diagram of this parallel information transmission technology is shown in Fig. S8. Suppose an array with five sensors work at different bias current regions. We can obtain five different images under the same beam of light illumination.

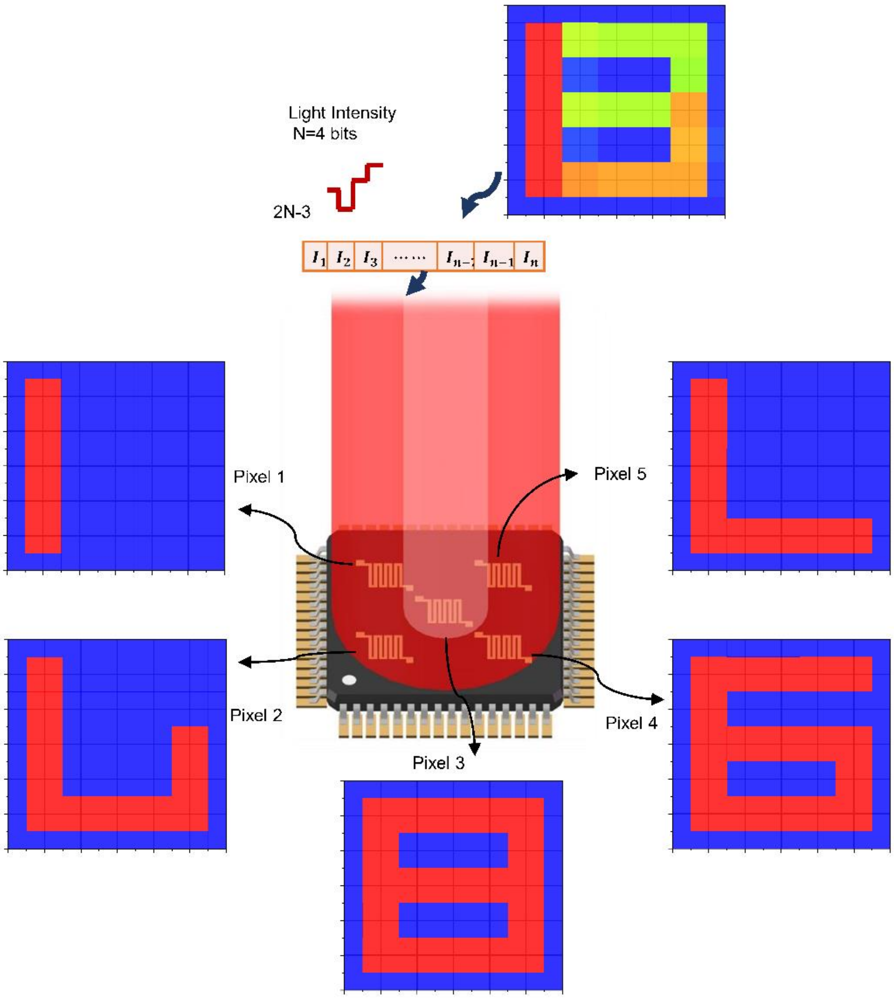


**Figure S8 Schematic diagram of parallel information transmission in memlogic arrays**. The information, which contained N=4 different intensity of light, is detected simultaneously by five memlogic sensor at different bias current. Then, five different images are obtained by five sensors, respectively.

## 10. Schematic diagram of ANN memlogic array

Our infrared memlogic device can be used for more complex and intelligent applications, such as Artificial Neural Networks (ANN). Figure S9a is a simplified diagram of a software-based ANN model, consisting of multiple neurons connected to each other through adjustable connection weights $W_i$. The output $Y$ depends on the weighted sum of inputs from preceding neurons and needs to accumulate to a certain threshold before outputting. Our device can be used for hardware implementations of ANN, as shown in the Fig. S9b and Fig. S9c. In our system, the output of the neural network can be expressed as $V_{out} = f(\sum R_i I_i)$, where the weights $W_i$ are represented by the light-responsive resistance $R_i$ of superconducting nanowires. Since the light-responsive resistance $R_i$ of superconducting nanowires exhibits highly nonlinear behavior, it can be controlled by adjusting bias current, light intensity, and temperature. We provide several methods for controlling the weight resistance $R_i$:

1. Independent bias current source control: Each device has an independent current source, and when the devices operate with different bias currents, their responses to light also vary, as shown in Fig 4.

2. Devices in the same column share the same bias current, but each device has an independent gate voltage to control the adjacent superconducting nanowires, similar to MIT's Tron[8].

3. Devices in the same column share the same bias current, but each device has independent control over temperature using metal heating wires, similar to MIT's htron[2].

The diagram depicted in the Fig. S9b illustrates a schematic representation of an Artificial Neural Network (ANN) that relies on bias current modulation. The following readout circuitry employs CMOS-based adders and comparators, facilitating the aggregation of weighted products derived from the light responses. The output is produced when a predetermined threshold voltage is attained.

We can also use localized temperature to adjust the weight of each pixel. In this scenario, each column of unit devices is connected in series and supplied with the same current ($I_{bias}$). The output voltage is the sum of voltages across all devices. Each device has its own separate control current or voltage to generate localized hotspots, thereby individually heating each unit device. This allows for the independent control of the critical current $I_c$ and $I_r$ of each unit device, effectively adjusting their weights.

Furthermore, as discussed in Fig. 6, our device, when operating at a specific bias current, exhibits a threshold response to light. It produces a substantial response only when the light intensity surpasses this threshold. Consequently, our hardware design encompasses adjustable weights, threshold responses, and summation operations, which constitute the foundational components of an artificial neural network (ANN).

Moreover, an intriguing aspect is that if each unit device possesses a distinct normal-state resistance (achievable through minor adjustments in device length during fabrication), we can discern which unit device in a column responds to light using just one readout voltage, denoted as *V1*. Conversely, traditional array detectors would necessitate at least N readout circuits to individually assess the voltages of N devices in the same column and pinpoint the specific unit device.

Our device also boasts wavelength tunability and scalability. We manipulate light through metamaterial and metasurface technologies. Our device incorporates metamaterial technology, enabling us to finely adjust its resonance response wavelength. As elucidated in supplementary Note 3, by manipulating the device's geometric parameters, we can customize the resonance wavelength to our desired band. Consequently, we can set different response wavelengths for each unit device. In the scenario where a sizable light spot simultaneously illuminates all the devices, only those devices resonating with the incident light will respond and yield an output. Therefore, our device can directly extract spectral information using a single voltage readout circuit. Furthermore, by integrating metasurface technology, we can spatially segregate light of varying color wavelengths, directing them onto distinct unit devices. This enhances light utilization efficiency and diminishes crosstalk.

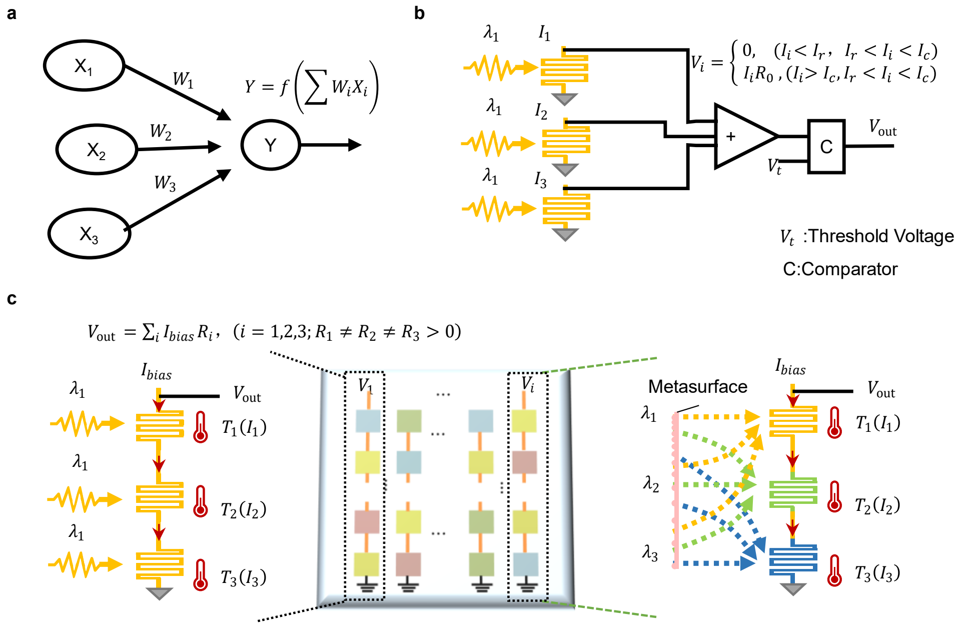


**Figure S9 Schematic diagram of ANN arrays**. **a** The Schematic diagram of software-based Artificial Neural Network (ANN). **b** The schematic diagram illustrates a superconducting nanowire ANN array with independent bias currents at each pixel, allowing for the adjustment of each pixel's weight. The output voltage is dependent on the summation of voltages from each pixel and the threshold voltage. **c** The Schematic diagram of superconducting nanowire ANN array with the same bias current at each pixel, but different local temperature at each pixel to adjust weight. The diagram on the left portrays each pixel designed to function at an identical operational wavelength, while the diagram on the right illustrates each pixel having a distinct detection wavelength. However, by designing the normal state resistance of each device differently, it becomes possible to infer the specific detected wavelength and spatial position information from the read voltage.

## 11. **Potential application of memlogic arrays**

The following diagram illustrates our device's capacity to emulate the focusing and memory functions of the human eye. In a scenario with a uniform light intensity field, such as the three letters "FDU" emitting light with equal intensity, we focus on distinct information, resulting in varied reading and storage outcomes. As depicted in our experiment (Fig. S10), when letters share the same light intensity, our devices initially operate at identical bias currents and generate indistinguishable signals. However, if our devices have undergone training, akin to the way our brains prioritize specific points in a scene and direct our gaze to those points (by increasing the bias current in a particular region of the array to attain a bistable state with memory, biasing the detector in the "FD" region to the "D" current region), this enhances the perception and memory of the region of interest, mimicking the human eye.

Even over time, our brains retain memory of the area of interest (the letters "FD") while selectively disregarding other details in the same scene (like the letter "U" with bias current in region C). The experiment validates that our detector possesses a

focusing and memory capability akin to that of the human eye. This ability can serve as preprocessing for visual information, enabling the detector to selectively store target information, filter out superfluous data, leading to reduced energy consumption, preserved storage space, and enhanced efficiency in subsequent, more intricate computational tasks.

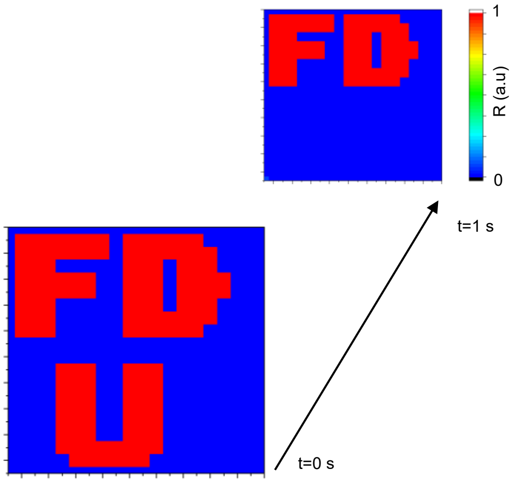


**Figure S10 Emulate the focusing and memory functions of the human eye by memlogic array.** The "FD" and "U" pixels were operated at different bias currents, with the "FD" pixel of the sensor being operated in a bistable state, exhibiting memory functionality.

Moreover, even at the individual device level, we can perform some simple image preprocessing functions. Simply convert a two-dimensional image into a one-dimensional array input to a laser, and then transmit it to the detector (Fig. S11). The signal perceived by the detector will filter out some noise points, as shown in the diagram below. At this point, our device operates under a constant current, and its noise filtering principle is illustrated in fig. 6. By filtering out noisy images, subsequent image recognition algorithms can reduce the number of training computations, decrease power consumption, and improve accuracy, as demonstrated by many researchers[9]. If the array was made, this process will be more efficiency.

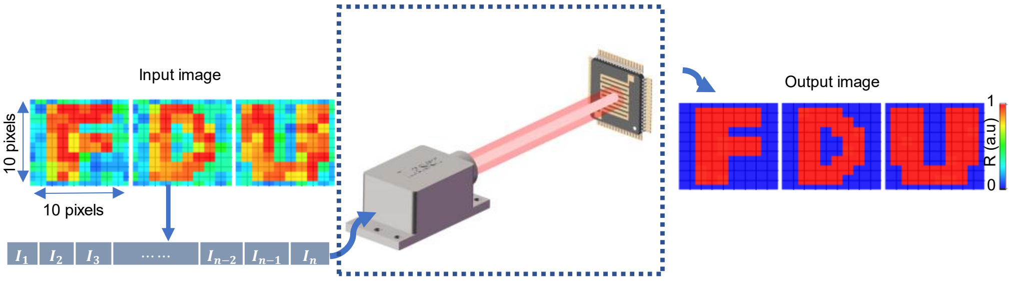


**Figure S11 Preprocessing the image in-sensor**. The memlogic sensor has the function of filter.

## 12. Comparison with others similar devices

**Table S1 Comparison with others similar device**

| Device | Sensing Wavelength | Responsivity | D* (Jones) | Memory Storage | Computing | Reconfigurable | Electrical speed | Optical speed | Power density | T (K) | Ref |
|---|---|---|---|---|---|---|---|---|---|---|---|
| **SNSPD[10]** | 10~29 μm | single photon | ~$10^{17}$ | × | × | × | √ | / | / | 0.1 | Ref.S10 |
| **Josephson+SNSPD[11]** | 0.78 μm | single photon | / | √ | neuromorphic | √ | 10 MHz | 250 ns ~5 ms | / | 0.8 | Ref.S11 |
| **Htron[2, 12]** | × | × | × | √ | × | × | 10 ns | × | ~$10^4$ nW μ$m^{-2}$ | 3 | Ref.S2,12 |
| **Josephson[13]** | × | × | × | √ | neuromorphic | √ | 100 GHz | × | ~1 nW μ$m^{-2}$ | 4 | Ref.S13 |
| **Ntron[8, 14]** | × | × | × | × | Logic | × | ~1 ns | × | ~80 nW μ$m^{-2}$ | 4.2 | Ref.S8,14 |
| **Superconducting Nanowire spiking element[15, 16]** | × | × | × | / | neuromorphic | √ | ~20 ns | × | ~0.0012 nW μ$m^{-2}$ | 4.2 | Ref.S15,16 |
| **Superconducting Bistability device** | **12.2 μm** | **~7.4 × $10^8$ V $W^{-1}$** | **1.2×$10^{14}$** | **√** | **Logic** | **√** | **~2 μs** | **~5 ms** | **60 nW μ$m^{-2}$** | **6.5** | **Our work** |
| **Superconducting Bistability[17]** | × | × | × | √ | × | × | × | × | ~60 nW μ$m^{-2}$ | 9.2 | Ref.S17 |
| **HgCdTe (MCT)[18]** | 10.6 μm | 0.15 A $W^{-1}$ | 2×$10^9$ | × | × | × | × | 3 ns | / | 197 | Ref.S18 |
| **PCM[19]** | × | × | × | √ | × | × | 40 ns | | 5 x $10^7$ nW μ$m^{-2}$ | 300 | Ref.S19 |
| **black phosphorus[20]** | 3.1 μm | 35 mA $W^{-1}$ | / | √ | neuromorphic | √ | / | 1~200 ms | / | 300 | Ref.S20 |
| **2D van der Waals heterostructures[21]** | 4.6 μm | 1.13 mA $W^{-1}$ | 9.6×$10^8$ | √ | neuromorphic | √ | / | 2.3 μs | / | 300 | Ref.S21 |

## 13. Reference


1. Golovashkin, A. I. et al. The Optical properties of niobium. *Soviet Physics Jetp* **29**, 27-34 (1969).
2. Baghdadi, R. et al. Multilayered Heater Nanocryotron: A Superconducting-Nanowire-Based Thermal Switch. *Physical Review Applied* **14,** 054011 (2020).
3. Heath, R. M. et al. Nanoantenna Enhancement for Telecom-Wavelength Superconducting Single Photon Detectors. *Nano Letters* **15**, 819-822 (2015).
4. Castilla, S. et al. Plasmonic antenna coupling to hyperbolic phonon-polaritons for sensitive and fast mid-infrared photodetection with graphene. *Nature Communications* **11.1,** 4872 (2020).
5. Lee, D. et al. High sensitivity bolometers based on metal nanoantenna dimers with a nanogap filled with vanadium dioxide. *Scientific Reports* **11,** 15863(2021).
6. Skocpol, W. J. et al. Self‐heating hotspots in superconducting thin‐film microbridges. *Journal of Applied Physics* **45**, 4054-4066 (1974).
7. Dane, A. et al. Self-heating hotspots in superconducting nanowires cooled by phonon black-body radiation. *Nature Communications* **13** , 5429 (2022).
8. McCaughan, A. N. A superconducting-nanowire three-terminal electrothermal device. *Nano Letters* **14**, 5748-5753 (2014).
9. Zhou, F. et al. Optoelectronic resistive random access memory for neuromorphic vision sensors. *Nature Nanotechnology* **14**, 776-782 (2019).
10. Taylor, G. G. et al. Low-noise single-photon counting superconducting nanowire detectors at infrared wavelengths up to 29 μm. *Optica* **10,** 1672-1678 (2023).
11. Khan, S. et al. Superconducting optoelectronic single-photon synapses. *Nature*

*Electronics* **5**, 650-659 (2022).

12. Butters, B. A. et al. A scalable superconducting nanowire memory cell and preliminary array test. *Superconductor Science and Technology* **34,** 035003 (2021).
13. Schneider, M. L. et al. Ultralow power artificial synapses using nanotextured magnetic Josephson junctions. *Science Advances* **4**, e1701329 (2018).
14. Zheng, K. et al. Characterize the switching performance of a superconducting nanowire cryotron for reading superconducting nanowire single photon detectors. *Scientific Reports* **9,** 16345 (2019).
15. Toomey, E. et al. Superconducting Nanowire Spiking Element for Neural Networks. *Nano Letters* **20**, 8059-8066 (2020).
16. Toomey, E. et al. Design of a Power Efficient Artificial Neuron Using Superconducting Nanowires. *Frontiers in Neuroscience* **13**, 933 (2019).
17. Buh, J. et al. Control of switching between metastable superconducting states in $\delta$-MoN nanowires. *Nature Communications* **6**, 10250 (2015).
18. https://www.thorlabs.com/newgrouppage9.cfm?objectgroup_id=11319
19. Wu, X. et al. Novel nanocomposite-superlattices for low energy and high stability nanoscale phase-change memory. *Nature Communications* **15,** 13 (2024).
20. Lee, S. Programmable black phosphorus image sensor for broadband optoelectronic edge computing. *Nature Communications* **13**, 1485 (2022).
21. Wang, F. et al. A two-dimensional mid-infrared optoelectronic retina enabling simultaneous perception and encoding. *Nature Communications* **14** , 1938 (2023).
